# Capillary Gradient Gel Electrophoresis

**DOI:** 10.3390/gels12010029

**Published:** 2025-12-30

**Authors:** Andras Guttman, Felicia Auer

**Affiliations:** 1 Horváth Csaba Memorial Laboratory of Bioseparation Sciences, Research Center for Molecular Medicine, Faculty of Medicine, University of Debrecen, 4032 Debrecen, Hungary; 2 Translational Glycomics Research Group, Research Institute of Biomolecular and Chemical Engineering, University of Pannonia, 8200 Veszprem, Hungary; auer.felicia@mk.uni-pannon.hu

**Keywords:** capillary electrophoresis, pore-size gradient gel, sieving matrices, proteins, nucleic acids

## Abstract

In the last half-century, capillary gel electrophoresis (CGE) became a versatile and high-performance analytical platform for the separation of complex biomolecular mixtures featuring rapid separations, high efficiency, and small sample consumption. Integrating a pore-size gradient mechanism in CGE makes it possible to achieve enhanced selectivity of polyionic macromolecules such as SDS-proteins and nucleic acids. This review provides a comprehensive overview of the theoretical foundations and operational principles of capillary pore-size gradient gel electrophoresis (CGGE), including the physicochemical basis of gradient formation, the influence of pore-size distributions on analyte mobility, and the challenges of generating stable, reproducible gradients in narrow-bore capillaries. Instrumental considerations such as capillary surface treatment, gradient filling and polymerization strategies, temperature and voltage control, detection modalities, and method-development frameworks are discussed in detail, emphasizing their critical impact on analytical performance and reproducibility. Key application areas in bioanalytical chemistry are highlighted, covering nucleic acid analysis and peptide/protein characterization. CGGE offers unique analytical advantages where fine molecular discrimination, tunable selectivity, and high resolution in a broad molecular weight range are required.

## 1. Introduction

The field of electrophoresis has undergone significant transformations since the groundbreaking work of Arne Tiselius in 1937 [1], culminating in the development of moving-boundary electrophoresis to separate serum proteins that earned him the Nobel Prize. To alleviate convective transport-related issues, gel-based systems were developed in slab formats using agarose or cross-linked polyacrylamide matrices, which provided the foundation for high-resolution analysis of polyionic macromolecules [2]. Traditional slab gel-based techniques, such as agarose and polyacrylamide gel electrophoresis (PAGE) as well as sodium dodecyl sulfate-PAGE (SDS-PAGE), became basic tools in practically all biochemistry and molecular biology laboratories for size-based separation of DNA/RNA and SDS-proteins [3]. However, the slab gel approach has multiple limitations, including extensive hands-on labor, long analysis times, poor quantitation accuracy due to staining variability, and lack of automation. The advent of capillary electrophoresis (CE) introduced by Jorgenson and Lukacs [4] in the early 1980s revolutionized the landscape by miniaturizing separations into narrow-bore fused-silica capillaries (typically 50–100 μm inner diameter), enabling high-voltage applications, rapid heat dissipation, and on-column detection for enhanced efficiency and reproducibility. The same year, Hjerten reported on the use of gel-filled capillaries for biopolymer separation [5], establishing the field of CGE as one of the important subsets of CE incorporating sieving matrices like cross-linked gels or entangled polymers within the narrow-bore tubing to facilitate molecular sieving [6]. This approach is particularly effective for the separation of large biomolecules with very similar mass to hydrodynamic volume ratios that do not separate well in free-solution CE modes like capillary zone electrophoresis (CZE). Early CGE applications focused on single-base resolution oligonucleotide separation [7] and protein sizing [8], with cross-linked polyacrylamide gels polymerized in situ within the separation capillary. However, bubble formation and limited column longevity prompted the shift to replaceable linear polymers in the 1990s, improving practicality [9], making possible automated capillary array-based DNA sequencing [10]. While conventional CGE employs homogeneous pore-size polymer networks, gradient gel systems have long been recognized as powerful tools in slab-gel electrophoresis due to their ability to provide continuously varying sieving characteristics [11]. Consequently, pore-size gradients enable fine control over electrophoretic mobility, improving the resolution of analytes with only subtle differences in molecular weight (MW) and/or conformation. In other words, gradient gel electrophoresis adds another layer of selectivity by introducing spatial or temporal variations in conditions—such as the size of the pores—along the separation path. However, translation of the benefits of gradient gels into capillary format introduces unique challenges, including controlled polymerization in confined geometries, gradient stability, and reproducibility. Capillary adaptations, referred to as CGGE in this review, represent a hybrid approach that unites the miniaturization, high efficiency, and automation potential of CE with the enhanced selectivity and dynamic range of gradient gel systems. Recent studies have demonstrated the feasibility of gradient-based systems in capillary and microchip formats, reporting applications ranging from DNA fragment extraction, analysis, and mutation detection to peptide profiling and synthetic polymer characterization [12,13,14,15]. As analytical demands continue to grow in genomics, proteomics, biopharmaceutical development, and materials science, gradient-based separation strategies offer new opportunities for tunable selectivity and improved resolution in capillary electrophoresis platforms.

This review delves into the fundamental principles of CGGE, methodological components (including sieving matrices, coatings, detection, and instrumentation), applications in biomolecular analysis, as well as advantages and limitations. By leveraging pore-size gradients, CGGE addresses the need for high-resolution separations of complex biological samples, bridging traditional electrophoresis with cutting-edge analytical demands from basic research to industrial applications.

## 2. Fundamental Principles of Capillary Gradient Gel Electrophoresis

### 2.1. Physical and Chemical Basis of Separation

Similar to constant pore-size (isometric) capillary gel electrophoresis, CGGE also operates on the core tenets of size-based electrophoretic migration, i.e., charged analytes move through a sieving matrix under the influence of an electric field with different mobilities [16]. However, the gradient component introduces dynamic pore size conditions during migration, enhancing the separation selectivity on one side for close structural and size variants, and on the other side for samples with broad MW differences in a single run. Thus, the fundamental separation mechanism in CGGE combines electrophoretic mobility with size-dependent sieving in a nonuniform polymer matrix.

The electrophoretic mobility of the analyte components is a function of the sieving matrix concentration (T_%_) and the retardation coefficient K_R_, as delineated by the Ferguson Equation (1) [17]. By plotting the logarithmic electrophoretic mobility vs. gel concentration, one can readily determine the free solution mobility (μ_0_) of the solute molecule and the retardation coefficient (K_R_), aiding matrix optimization [3].

log μ = log μ_0_ − K_R_T_%_ (1)

As the analyte molecules migrate through the gradient, they encounter progressively smaller pores, resulting in position-dependent mobility. Larger molecules experience reduced mobility earlier in the gradient, whereas their smaller counterparts penetrate deeper before being substantially retarded. Therefore, the T_%_ term is defined by the gradient as in position x along the axis of the column as follows:

(2) $$T_{\%}=T_{\%}^{I}+\frac{{dT}_{\%}^{O-I}}{dx}x$$

where $T_{\%}^{I}and$ $T_{\%}^{O}$ are gel concentrations at the capillary inlet and outlet sides, respectively, and x is the distance along the capillary. Combining Equations (1) and (2) results in the mobility equation in CGGE:

(3) $$\log\mu =log\mu_{0}-K_{R}(T_{\%}^{I}+\frac{{dT}_{\%}^{O-I}}{dx}x)$$

The local electric field of E(x) also changes with the gradient, due to the decreasing conductivity of the higher concentration gel section.

(4) $$E(x)=\frac{J}{\sigma (x)}$$

where J is the current density and σ(x) is the local conductivity at position x. This phenomenon causes higher potential drops at the higher gel concentration regions also influencing the electrophoretic mobility of the migrating species.

The interplay between electrophoretic mobility, polymer concentration, as well as the distribution of pore size and electric field strength defines the analytical performance of CGGE. Therefore, understanding these factors is crucial for optimizing gradient design and achieving high-resolution separations.

### 2.2. Background of Gradient Gels

Gradient gels have been widely used in slab-gel electrophoresis for decades, because the continuous change in gel concentration—or more generally, pore size—introduces a spatially modulated sieving environment that enhances molecular-level resolution across broad size ranges [18]. In polyacrylamide gradient gels, a typical configuration involves a low-percentage region of large pores transitioning to a high-percentage region of progressively smaller pores, allowing separation of analytes that would otherwise overlap on a single-percentage (isometric) gel. This concept is well established in traditional protein and nucleic acid analysis, where pore-size gradient slab gels enable the differential migration of species that vary only subtly in molecular size. For example, polyacrylamide gradient gels in the 4–20% acrylamide concentration range have long been used to resolve proteins and DNA fragments with challenging size overlaps or conformational heterogeneity [19,20].

The formation of pore-size gradients can be achieved either by chemical polymerization—typically initiated by ammonium persulfate (APS) and N,N,N′,N′-tetramethylethylenediamine (TEMED) [2] or via photopolymerization approaches [21]. While chemical polymerization remains the most widely used technique, photoinitiated polymerization offers advantages such as high precision spatial control and reduced oxygen inhibition. Practical gradient formation, whether in tubes or in slab casting chambers, can be performed using dedicated gradient mixers or through simplified two-solution layering approaches, provided that convective mixing is minimized to maintain linearity of the gradient [18].

The analytical benefits of gradient gels are thus twofold: they enable high-resolution separation of species spanning broad MW ranges, and they significantly improve discrimination among closely related molecular entities, i.e., properties that form the conceptual basis for implementing pore-size gradient gels into narrow-bore capillaries. Nucleic acid analysis benefits the application of gradient principles to detect subtle differences unattainable in uniform conditions, e.g., for mutation detection, while in the case of proteins, it allows size separation in a wide MW range.

### 2.3. Capillary Format Adaptations for Pore-Size Gradient Gels

Adapting gradient gel systems to a capillary format introduces substantial engineering and materials challenges. Capillaries used for electrophoresis are most commonly constructed of fused silica, which provides a mechanically robust yet optically transparent structure suitable for on-capillary detection. However, at higher pHs, the charged silanol groups on the bare fused silica surfaces can induce strong electroosmotic flow (EOF), driven by the zeta potential at the inner wall, and may also cause undesired analyte–wall interactions. Therefore, internal surface coatings (permanent or dynamic) are often required to suppress EOF, reduce protein adsorption, and stabilize the polymer matrix [22].

Creating a stable and reproducible gradient within a capillary is significantly more complex than in slab-gel systems. Maintaining a continuous concentration gradient in narrow internal diameter capillaries (typically ranging from 25 to 100 µm), requires precise control of monomer-cross-linker introduction, diffusion, and polymerization kinetics. Several micro-analytical electrophoresis publications highlight challenges when making cross-linked polyacrylamide gels in capillaries, including bubble formation, incomplete polymerization, nonuniform pore-size distribution, and difficulties with degassing the monomer solutions [23]. Non-cross-linked (entangled) polymer matrices (Table 1), such as linear polyacrylamide, dextran, derivatized celluloses, pullulan, and polyethylene oxide, offer easier handling because they are replaceable and do not require in-capillary polymerization [24]. However, establishing a true and stable gradient with these physical gels is more difficult because such systems tend to re-equilibrate in time by diffusion. Thermal constraints further complicate the establishment of such gradient gels in capillary format. Albeit narrow bore tubings support the application of high electric field strengths to increase separation speed but also generate Joule heat. Without sufficient heat dissipation, temperature gradients can distort the intended polymer gradient, modify viscosity, and introduce convection. These effects are well-documented challenges in CE, even in homogeneous sieving matrices [25], and are amplified in gradient-based systems. To alleviate these issues, novel engineering solutions have been introduced to preserve gradient fidelity during operation [12,26].

### 2.4. Gels and Sieving Matrices

The sieving matrix is central to the performance of CGGE, providing the proper reticulations while allowing replaceability (Table 1). It is important to note that for gradient applications, matrices must withstand chemical/thermal stresses. Gels in the early 1990’s, polymerized in-capillary with acrylamide/bis-acrylamide, offered high resolution but suffered from bubble formation and short lifespans (<10 runs) [26,27]. Replaceable polyacrylamide at 3–6% concentrations, on the other hand, resolved proteins across wide MW ranges in <20 min, with low viscosity for easy replenishment [28,29]. Polysaccharide-based matrices like dextran [30], pullulan [31], hydroxypropylcellulose (HPC) [32], and hydroxyethylcellulose (HEC) [33] all feature low UV absorbance and moderate viscosity, suitable for uncoated capillaries. Polyethylene oxide (PEO) [34] and polyvinyl alcohol (PVA) [35] suppress EOF, which proved beneficial in DNA and SDS-protein separations. Temperature-responsive matrices, such as PEO–PPO–PEO triblock copolymers or phospholipid pseudogels, self-assemble and adjust pore sizes with heat [36]. Transiently cross-linked isometric borate-dextran matrices were successfully applied in SDS-protein analysis [37].

## 3. Instrumentation

### 3.1. Instrumental Architecture and System Components

CGGE builds upon the established instrumentation of CE, incorporating precise high-voltage control, temperature-stabilized capillary environments, automated sample handling, and sensitive optical detection. Modern CE systems are engineered to sustain stable electric fields in narrow-bore capillaries while minimizing the effect of Joule heating and preserving the physicochemical integrity of polymer matrices. As described in several comprehensive reviews, advancements in CE instrumentation have included enhanced mechanical robustness of the capillary holding cassette, improved high-voltage ramping profiles, and superior thermalmanagement hardware [38,39].

**Figure 1:**
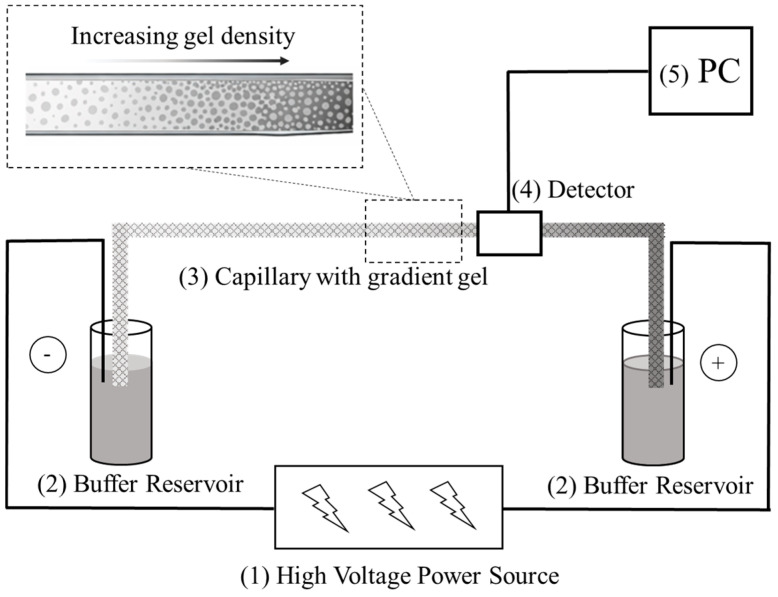
Basic setup of a CGGE system. (1) high voltage power supply; (2) buffer reservoirs containing the background electrolyte; (3) separation capillary with the pore-size gradient gel; (4) optical detection system (UV or LIF); (5) PC (data acquisition/processing computer).

A standard CGGE setup includes a high-voltage power supply (commonly 10–30 kV), a thermostatted capillary cassette, precise pressure modules, and most frequently either UV or fluorescence detection, as the schematics depict in Figure 1. High-voltage stability is crucial because small variations in field strength can produce significant shifts in migration behavior within gradient gels, where mobility is already axial position-dependent (Equation (4)). Temperature control is equally important; capillaries are typically confined in liquid cooling systems or mounted in aluminum or polymer composite cartridges with active Peltier cooling to ensure proper heat dissipation and uniform thermal conditions along their length [6].

Automation has improved significantly through the introduction of autosamplers, programmable rinse cycles, and integrated capillary conditioning routines, enabling high-throughput operation and reproducible quantitative analyses. Multi-capillary systems further expand throughput, although implementing gradient matrices in arrays presents additional challenges in ensuring uniform gel filling in each capillary and gradient reproducibility.

### 3.2. Sample Introduction Techniques

Sample introduction strongly influences CGGE performance [40]. Pressure injection is preferred for routine work due to its reduced susceptibility to analyte mobility differences, but not always possible due to the high viscosity gel matrix in the column. In addition, the parabolic flow profiles inherent with hydrodynamic injection were considered to cause dispersion. Electrokinetic injection, although not gel viscosity dependent, may distort the initial sample plug shape and introduce bias for analytes with different electrophoretic mobilities, effects that are amplified in gradient matrices [41].

Available literature demonstrates that optimized injection parameters (pressure, time, capillary ID) are critical for maintaining sharp and symmetric zones, even in conventional isometric CGE [6]. In gradient systems, the injection zone must be placed at the low-concentration (large-pore) region of the gradient to avoid premature sieving (Figure 1). Advanced pre-concentration techniques such as field-amplified sample stacking (FASS) [42], sweeping, and dynamic pH junctions [43,44] have been successfully integrated into polymer-based capillary systems and may offer potential sensitivity enhancement even for pore-size gradient systems.

### 3.3. Thermal Management and Control of Joule Heating

Joule heating is a major concern during the use of polymer-filled capillaries [45]. Even modest electric currents can create temperature gradients which—because of the thermally sensitive nature of polymer networks—may alter pore size, viscosity, and local electrophoretic mobility. Temperature gradients can also generate convection and disrupt the intended gradient geometry for non-cross-linked polymers. Rathore emphasized that CE performance depends strongly on efficient heat dissipation, and showed that modern systems employ optimized Peltier blocks, improved thermal coupling materials, and low-conductivity buffers to maintain system stability [46]. In CGGE, thermal control is even more critical to assure that the axial concentration gradient remains intact, and changes in temperature do not compress or expand the gradient.

Routine mitigation strategies include operating at reduced ionic strength and employing low-conductivity buffer systems to limit Joule heating, while applying moderate field strengths with controlled voltage ramping to avoid abrupt thermal gradients. In addition, maintaining tight temperature regulation—typically within ±0.1 °C—and continuously monitoring the electrophoretic current provide effective means of detecting and preventing gradient deformation during separation.

### 3.4. Detection Strategies and Data Acquisition

Optical detection is integral to CGGE. UV absorbance (e.g., 214 or 280 nm for proteins and 260 nm for nucleic acids) remains the most widely used method due to its simplicity and compatibility with native and denaturing conditions. High-quality CGGE data acquisition requires stable baselines, noise-free optical paths, real-time monitoring of current and temperature, and reliable migration-time alignment algorithms. However, polymer matrices scatter light more strongly than free-solution CE background electrolytes, so optical alignment and capillary window preparation are critical. Fluorescence detection—particularly laser-induced fluorescence (LIF)—has emerged as an important alternative because of its superior sensitivity, often yielding two orders of magnitude better detection limits in CE-based assays [47]. Mass spectrometric (MS) detection is a new expanding field, though coupling sieving matrices to MS remains challenging because polymers may interfere with ionization. While CE-MS has been thoroughly developed for free-solution electrophoresis, CGE–MS is still in its infancy. Improvements in sheath-flow nanospray interfaces and polymer-compatible ionization sources may eventually allow routine integration, as suggested by recent CGE–MS interface studies [48]; however, these are not readily applicable to CGGE-MS.

## 4. Method Development

### 4.1. Technical Challenges in Gradient Matrix Preparation, Capillary Filling, and Polymerization

**Figure 2:**
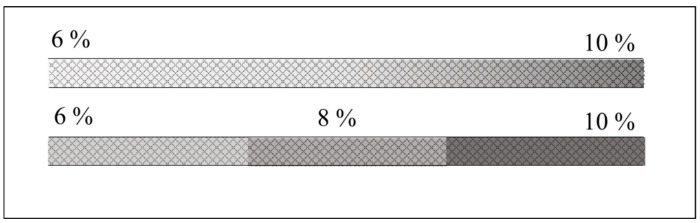
Schematics of continuous pore-size (**upper**) and step gradient (**lower**) gel filled capillary columns.

Creating a stable and reproducible axial pore-size gradient inside a capillary remains one of the most technically challenging aspects of CGGE, regardless of whether it is a step or a continuous gradient (Figure 2).

Table 2 summarizes the challenges associated with the fabrication of capillary pore-size gradient gels. Traditional cross-linked polyacrylamide is difficult to uniformly polymerize inside narrow bore capillaries. Oxygen inhibition, bubble formation, and differential polymerization kinetics along the capillary length are well-known limitations [15]. Studies on polymerization in microchannels have shown that photoinitiated polyacrylamide formation can improve spatial uniformity and reduce polymerization gradients, making it a promising technique for gradient gel fabrication [14]. Schwarz and coworkers reported on the preparation of highly condensed polyacrylamide gels, including step gradient gels [26]. Wang and Beale utilized a micro-chamber system for casting step gradient gels [12]. After mixing the polymerization reaction ingredients, the micro-chambers were filled with the respective polymer solution, allowing them to cast up to six gradient steps.

For replaceable, non-crosslinked matrices (i.e., physical gels, e.g., linear polyacrylamide, polyethylene oxide, dextran), diffusion-controlled gradient formation is possible but susceptible to temporal broadening. To establish a stable gradient, two polymer solutions of different concentrations may be loaded sequentially using controlled-pressure filling; alternatively, gradient-maker syringe assemblies can be adapted from slab-gel methodologies. For uncommon sieving systems, Miksík et al. reviewed the rheological properties, viscosity–concentration relationships, and CE applicability even for uncommon gel types [49].

Reproducibility in CGGE is strongly influenced by the preparation of both the inside surface of the capillary and the gradient matrix. A key requirement is the use of appropriate capillary surface treatments—either dynamic polymer coatings or permanent covalent modifications—to suppress undesired electroosmotic flow and minimize analyte–wall interactions, both of which can otherwise introduce significant migration-time variability. Equally important is the complete removal of dissolved gases from monomer or polymer solutions. Ensuring bubble-free filling of the capillary is, therefore, essential for maintaining the continuity of the gradient and the uniformity of the electromigration path. For cross-linked gradient gels, precise control of polymerization reaction conditions—including initiator concentration, reaction environment, and polymerization time—is critical to achieving the intended pore-size profile along the capillary. Finally, the long-term stability of the gradient matrix depends on proper storage under refrigerated or strictly isothermal conditions, as temperature fluctuations or prolonged idle periods can lead to gradual structural relaxation or diffusive smoothing of the gradient, thereby reducing run-to-run consistency.

### 4.2. Method Optimization and Quality Assessment

Method optimization in CGGE is intrinsically multi-parameteric, since separation performance is governed by the interplay of capillary geometry, gradient design, polymer matrix properties, and the applied electric field. Experience from isometric CGE shows that column length and internal diameter determine the available separation window, heat dissipation, and practical analysis time [6]. Longer effective lengths improve resolution but increase run time, whereas smaller internal diameters enhance heat transfer at the cost of higher backpressure during matrix loading and greater sensitivity to blockages.

The design of the axial gradient with its overall concentration range and slope is a CGGE-specific variable that must be fine-tuned to the size distribution of the sample mixture in hand. In pore-size gradient gels, the shape and linearity of the gradient directly control the relationship between migration distance and molecular size. Deviations from a smooth, linear profile quickly lead to loss of resolution or non-monotonic mobility behavior. In practice, this means that the preparation protocol (mixing of polymer solutions, filling dynamics, and any in-capillary polymerization) must be tightly controlled and standardized.

The polymer matrix itself represents another optimization challenge. Critical reviews of sieving matrices in microanalytical electrophoresis emphasize that polymer type, MW distribution, and concentration jointly dictate viscosity, ease of capillary loading, sieving strength, and robustness against shear [24]. For example, comparison between linear polyacrylamide and polydimethylacrylamide matrices in DNA analysis have highlighted the trade-offs between viscosity, ability to suppress electroosmotic flow, and long-term stability [50].

The electrical and thermal operating conditions must be optimized in parallel with the matrix gradient. Systematic studies on DNA and protein separations in polymer-filled capillaries show that resolution and reproducibility are highly sensitive to electric field strength, buffer composition, and temperature, because these parameters influence both electrophoretic mobility and polymer conformation [51]. In pore-size gradient gels, where mobility is intrinsically position-dependent (Equation (3)), small changes in the local electric potential drop (Equation (4)) or temperature change can translate into disproportionate shifts in band positions along the gradient. Analytical method-development frameworks for CGGE therefore requires the definition of acceptable operational ranges for voltage, temperature, and buffer ionic strength during robustness testing, and quantifying their impact on resolution and migration times. The reproducibility critically depends on how the capillary and gradient matrix are prepared and maintained. Work on CGE of DNA plasmid topoisomers, using unmodified fused-silica capillaries, showed that migration-time precision on the order of 1% (RSD) across more than 150 runs is achievable [52], but only when the gel preparation protocol, dye concentration, and capillary-conditioning steps are rigorously controlled. These findings mirror earlier observations in isometric polymer-matrix CE, where capillary conditioning (rinsing with base, acid, water, and fresh polymer), suppression of electroosmotic flow, and stable coating chemistry were identified as key factors minimizing day-to-day variability.

For CGGE, additional attention must be paid to preserving gradient integrity during storage and between runs, e.g., as mentioned above, by keeping pore-size gel filled capillaries under controlled temperature and avoiding extensive idle periods that could allow diffusive smoothing of the gradient. From a quality-assessment perspective, CGGE methods should be evaluated using the same fundamental metrics as other electrophoretic techniques—efficiency (theoretical plate numbers, N), resolution (Rs) between critical sample component pairs, peak symmetry (electromigration dispersion [53]), and migration-time precision—supplemented by gradient-specific diagnostics. These emphasize the importance of employing Design-of-Experiments (DoE) or Analytical Quality by Design (AQbD) approaches to map the design space and identify robust operating conditions that meet predefined analytical performance criteria. In CGGE, such studies can be complemented by routine checks of the gradient itself, for instance by monitoring the migration of a panel of MW standards across the capillary or by using fluorescent probes to visualize the effective gradient profile. Together, these strategies allow CGGE methods to achieve the level of robustness and traceability required for complex macromolecular analysis, potentially even for regulated applications.

## 5. Applications in Bioanalytical Chemistry

Capillary gel electrophoresis, including gradient gel formats where applicable, has become an important analytical platform in modern bioanalytical chemistry due to its ability to resolve complex mixtures of nucleic acids, proteins, peptides, complex carbohydrates and other biomolecules with high efficiency and minimal sample consumption [6]. The controlled sieving environment provided by polymer matrices, combined with precise thermal and electrical regulation, enables CGGE systems to address analytical challenges that are difficult to manage using free-solution capillary electrophoresis or isometric CGE. The versatility of these systems holds the promise to address significant applications across genomics, proteomics, biopharmaceutical analysis, and clinical research.

### 5.1. Nucleic Acids

Capillary gel electrophoresis has long been applied to the analysis of nucleic acids, particularly in the context of DNA sizing, conformational studies, and structural probing. The review by Holland and coworkers demonstrates [54] how polymer-based sieving matrices facilitate high-resolution separation of DNA fragments, hairpins, G-quadruplexes, and topological variants, and in what way subtle matrix–analyte interactions can be exploited to improve analytical sensitivity and selectivity. These principles extend directly to CGGE, where gradient-based pore structures can be engineered to discriminate among fragments differing by only a few nucleotides, even with the same chain length. Such capabilities are increasingly relevant in the quality assessment of therapeutic oligonucleotides—including antisense oligonucleotides, siRNA constructs, and chemically modified mRNA—where differentiation of truncated species, degradation products, or modification variants is critical for regulatory compliance and product characterization. Recent biopharmaceutical applications highlight the suitability of CE-based sieving for purity assessment and impurity profiling of these new therapeutic modalities [55].

**Figure 3:**
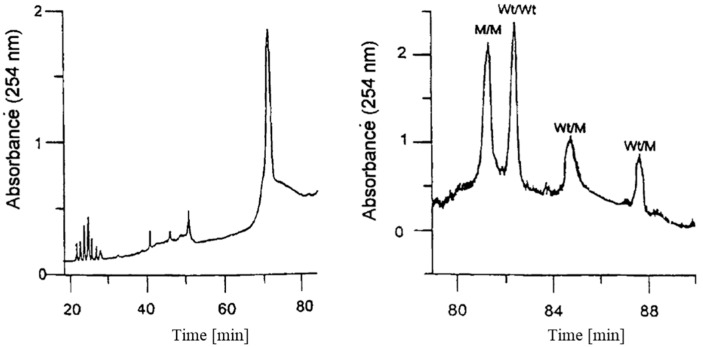
Separation of a point mutation in exon 11 of the cystic fibrosis gene (17171 G + A) with an isometric 6% constant pore size (**left panel**) and a gradient of 6 to 8% polyacrylamide gel (**right panel**). M—mutant, Wt—wild type. With permission from [15].

Righetti and Gelfi [15] reported on the fabrication of a porosity gradient gel to decompress the zones of homo- and heteroduplexes, which otherwise migrated in smeared and diffuse bands, by sequentially applying a series of step concentrations of the required porosity. The resulting gradient was uniform, in particular at the point of detection. Figure 3 shows the striking difference between the electrophoretic separation based on a point mutation in exon 11 of the cystic fibrosis gene (17171 G + A) with a 6% constant pore size (left panel) and a gradient of 6% to 8% (right panel) polyacrylamide gel, with an additional temperature gradient of 56–58 °C in both instances.

### 5.2. Proteins and Peptides

Protein analysis remains the other most established field for polymer-mediated capillary electrophoresis. Gradient gels can provide additional resolving power either for closely related protein isoforms, truncated variants, products bearing subtle post-translational modifications, or sample mixtures in broad size range. Wang and Beale [12] reported on the use of linear polyacrylamide gel gradient along the capillary column with sodium dodecyl sulfate detergent for the analysis of a peptide—protein mixture in the broad MW range of 6 to 97 kDa in less than 15 min.

Bhimwal et al. [56] recently published a comprehensive summary of the advances in CGE for protein separations, emphasizing its value in biopharmaceutical development for size heterogeneity analysis, glycoform discrimination, and stability testing. In monoclonal antibody characterization, sodium dodecyl sulfate capillary gel electrophoresis (SDS-CGE) has become the industry standard for monitoring degradation pathways, clipped species, and non-reduced structural variants due to its excellent reproducibility, automation potential, and compatibility with regulatory expectations. Gradient-based capillary approaches may further refine such analyses by tuning the sieving regime for increased resolution of species with minimal mass or conformational differences.

**Figure 4:**
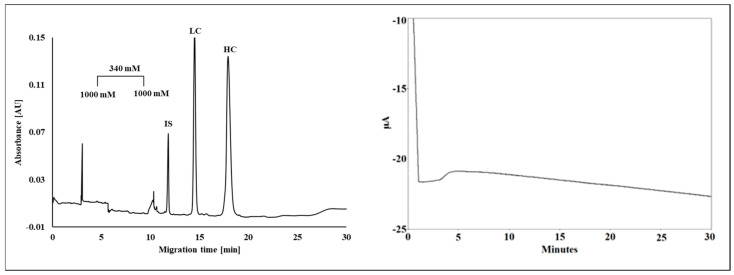
Temporal pore-size gradient SDS-CGE separation of the subunits of a therapeutic monoclonal antibody. The boric acid cross-linker concentration was increased from 340 mM to 1000 mM in the capillary in time. IS—internal standard, LC—light chain, HC—heavy chain. The right panel shows the corresponding change in the current trace. With permission from [53].

Instead of the traditional use of spatial pore-size distribution, Filep and Guttman [53] introduced the interesting concept of temporal gradient gels in SDS-CGE of proteins (Figure 4). Increasing the boric acid cross-linker concentration in time from 340 mM to 1000 mM changed the pore-size of the gel in the separation capillary, recognized by the current change (Figure 4, right panel). This approach also alleviated electromigration dispersion.

### 5.3. Emerging Directions in Microfluidic and Integrated Bioanalysis

Recent technological developments suggest that the integration of controlled-gradient sieving matrices into microfluidic CE systems may open new avenues in high-throughput and single-cell bioanalysis [57]. Microstructured and nanostructured polymer gels have been shown to facilitate separations with improved resolution and speed [58], particularly when combined with laser-induced fluorescence or electrospray ionization mass spectrometry detection [6]. Contemporary electrophoresis trends indicate a growing interest in platform miniaturization, multi-capillary arrays, and hybrid detection strategies, all of which enhance the analytical versatility of polymer-based separations [43]. Although CGGE in its strict form has been less frequently implemented on microchips, the underlying principles—finely tuned pore-size gradients, spatially controlled sieving behavior, and temperature-stabilized environments—align closely with the goals of next-generation microfluidic analytical systems.

## 6. Conclusions

Capillary gradient gel electrophoresis represents a technically demanding yet analytically powerful extension of isometric CGE, combining the benefits of high-efficiency capillary formats with the tunable selectivity of spatially or temporally structured polymer sieving environments. Gradient gels are most appropriate for the analysis of complex mixtures such as cell extracts and body fluids, especially if the sample components cover a wide molecular size range. As demonstrated throughout this review, the successful implementation of CGGE relies on an intricate balance between polymer chemistry, capillary surface engineering, gradient fabrication, and precise control of electrical and thermal conditions. Advances in polymer science, microfabrication, and capillary instrumentation have collectively enabled more reproducible gradient formation, reduced thermal artefacts, and enhanced detection capabilities, thereby overcoming several limitations that historically hindered widespread adoption of CGGE. The theoretical and practical principles discussed—ranging from pore-size gradient design and mobility modulation to capillary conditioning and method-development strategies—highlight that CGGE is uniquely suited for applications requiring fine molecular discrimination, such as the analysis of closely related nucleic acid fragments, protein isoforms, therapeutic oligonucleotides, and structurally complex macromolecular species.

Recent progress in microfluidic integration and high-sensitivity detection methods suggests that gradient gel-based capillary systems will continue to evolve, potentially enabling high-throughput via multiplexing and supporting even single-cell bioanalytical workflows. Although challenges remain, particularly in the routine fabrication of highly reproducible gradients and the integration of gradient matrices with mass-spectrometric detection, the evidence surveyed in this article underscores that CGGE has matured from a conceptual extension of slab-gel gradients into a promising analytical technology with distinct advantages. Continued developments in gradient engineering, instrument design and polymer materials are likely to further expand its applicability, especially with the help of artificial intelligence (AI) based gradient design. As bioanalytical demands grow increasingly complex—driven by protein biotherapeutics, single-cell analysis, mRNA technologies, and precision diagnostics—CGGE is poised to serve as a valuable and adaptable analytical tool capable of fulfilling these emerging challenges.

## Figures and Tables

**Table 1:** The most frequently used polymers in CGE with the option of generating pore-size gradients.

Polymer	Repeating Unit	Viscosity	UV Cutoff	Typical Use
Dextran (borate cross-linked)	(C_6_H_10_O_5_)_n_	Moderate to high (tunable)	Low	Tunable and replaceable sieving matrix for CGE; widely used for SDS-protein separations, biopharmaceutical analysis, and method development requiring adjustable sieving properties
Polyacrylamide (linear, LPA)	(C_3_H_5_NO)_n_	Low to moderate	Moderate	Classical replaceable sieving matrix in CGE for DNA and proteins; ease of replenishment, and routine separations
Cross-linked polyacrylamide	(C_3_H_5_NO)_n_ + cross-links	High	Moderate	High-resolution CGE tunable for wide MW ranges; preferred when enhanced sieving efficiency and improved resolution are required
Poly(vinyl alcohol) (PVA)	(C_2_H_4_O)_n_	Low	Low	Dynamic wall-coating polymer in CGE; used to suppress EOF and analyte–wall interactions, often combined with other sieving matrices
Hydroxyethyl cellulose (HEC)	Cellulose backbone with –CH_2_CH_2_OH substituents	Low to moderate	Low	Physical matrix in CGE; applied in DNA and protein separations where temperature-dependent viscosity control is beneficial
Poly(ethylene oxide) (PEO)	(C_2_H_4_O)_n_	Low to moderate	Low	Replaceable entanglement-based sieving matrix for CGE; suitable for DNA and protein separations with flexible tuning via polymer MW
Pullulan	(C_6_H_10_O_5_)_n_	Moderate	Low	Neutral polysaccharide sieving matrix in CGE; used for DNA and protein separations where low UV absorbance and mild polymer–analyte interactions are desired

**Table 2:** Technical challenges with the fabrication of capillary pore-size gradient gels.

Technical Challenge	Origin/Cause	Impact on CGGE	Reported Solutions
Gradient reproducibility	In-capillary gradient formation and polymerization	Run-to-run variability; limited robustness	Controlled polymerization protocols; degassing; surface-treated capillaries
Gel stability and lifetime	Mechanical stress and bubble formation in gradient gels	Short column lifetime; limited throughput	Optimized gel composition; pressure control; improved capillary coatings
Limited matrix replaceability	Cross-linked/high viscosity gradient gels	Reduced suitability for routine use	Step-gradient approaches; diffusion-based gradients; replaceable polymer systems
High viscosity of gradient gels	Dense sieving matrices	Difficult capillary loading; pressure requirements	Pressure-assisted filling; temperature-controlled loading
Gradient relaxation	Diffusion between gradient zones	Loss of gradient sharpness	Optimized gradient length; minimized equilibration time
Coupling to MS	Gel and detergent incompatibility with MS	No CGGE–MS implementation	Gel-free detection zones; partial gel removal; specialized CE–MS interfaces
Limited quantitative robustness	Gradient instability and detector constraints	Challenges in validation and quantitation	Temperature stabilization; internal standards; optimized detection conditions

## Data Availability

The data are available upon request from the authors.

## Abbreviations

The following abbreviations are used in this manuscript:

CGE	Capillary Gel Electrophoresis
CGGE	Capillary Gradient Gel Electrophoresis
EOF	Electroosmotic flow
LIF	Laser-induced fluorescence
MW	Molecular weight
MS	Mass spectrometry
SDS-CGE	Sodium dodecyl sulfate capillary gel electrophoresis
